# Development of a rapid, simple assay of plasma total carotenoids

**DOI:** 10.1186/1756-0500-5-521

**Published:** 2012-09-24

**Authors:** Michael Donaldson

**Affiliations:** 1 Hallelujah Acres, 1733 Cutler Way, Zillah, WA, 98953, USA

**Keywords:** Carotenoid, HPLC, Spectrophotometry, Assay method, Beta-carotene, Lycopene

## Abstract

**Background:**

Plasma total carotenoids can be used as an indicator of risk of chronic disease. Laboratory analysis of individual carotenoids by high performance liquid chromatography (HPLC) is time consuming, expensive, and not amenable to use beyond a research laboratory. The aim of this research is to establish a rapid, simple, and inexpensive spectrophotometric assay of plasma total carotenoids that has a very strong correlation with HPLC carotenoid profile analysis.

**Results:**

Plasma total carotenoids from 29 volunteers ranged in concentration from 1.2 to 7.4 μM, as analyzed by HPLC. A linear correlation was found between the absorbance at 448 nm of an alcohol / heptane extract of the plasma and plasma total carotenoids analyzed by HPLC, with a Pearson correlation coefficient of 0.989. The average coefficient of variation for the spectrophotometric assay was 6.5% for the plasma samples. The limit of detection was about 0.3 μM and was linear up to about 34 μM without dilution. Correlations between the integrals of the absorption spectra in the range of carotenoid absorption and total plasma carotenoid concentration gave similar results to the absorbance correlation. Spectrophotometric assay results also agreed with the calculated expected absorbance based on published extinction coefficients for the individual carotenoids, with a Pearson correlation coefficient of 0.988.

**Conclusion:**

The spectrophotometric assay of total carotenoids strongly correlated with HPLC analysis of carotenoids of the same plasma samples and expected absorbance values based on extinction coefficients. This rapid, simple, inexpensive assay, when coupled with the carotenoid health index, may be useful for nutrition intervention studies, population cohort studies, and public health interventions.

## Background

Diets high in fruits and vegetables and sufficient in all other essential nutrients are vital to excellent health. Fruits and vegetables are nutritionally dense sources of vitamins and minerals. They are the best dietary source of antioxidants and protective phytochemicals. One aspect of the 2010 Dietary Guidelines in the USA is to increase intakes of fruits and vegetables. A recent review of the human data on plasma carotenoids and health outcomes established a carotenoid health index and concluded that over 95 percent of the population of the USA has concentrations of total plasma carotenoids less than 2.5 μM, which puts them at moderate or high risk for negative health outcomes [1]. A measurement of total plasma carotenoids would give people objective personal feedback on how much risk they carry due to their intake, or lack of intake, of antioxidant-rich and carotenoid-rich fruits and vegetables.

Carotenoids are typically quantified by HPLC analysis. This specialized analysis is relatively expensive, time-consuming, and not easily used beyond the research laboratory. Each analysis takes approximately 20 to 30 minutes, though one method has a run time under 12 minutes [2], and a technician is needed to prepare blood extractions and to oversee the operation of the HPLC system, even when an autosampler is used. Each spectrophotometric assay takes 3 minutes when performed in a series of samples, and, if adapted to microwell format, could take even less time per sample. Supplies and solvents cost about $US 0.12 per spectrophotometric assay. The spectrophotometric assay uses less than 0.5 ml of solvent per assay, while the HPLC method uses 2 ml per minute of run time and additional solvents for the extraction procedure. The simpler spectrophotometric assay of carotenoids is faster and cheaper, yet it has been assumed to be less accurate compared to HPLC methods. The HPLC method also gives quantitative information about individual carotenoids, which cannot be done with a spectrophotometer.

Spectrophotometric analysis of carotenoids has been known and practiced since at least 1914, with the observation that carotenoids (carotins and xanthophylls and lipochromes in this older literature) could be extracted from serum by first treating the serum with alcohol and then with petroleum ether or another nonpolar solvent [3]. The process was further refined by Connor in 1928, but the color standard used was potassium dichromate [4]. The process has been much improved, but the basic technique is essentially the same in modern laboratories. However, in searching for published techniques for total carotenoids, none were found that showed the correlation of the spectrophotometric method with plasma standards of mixed carotenoids. Sayoun and coworkers [5] used a petroleum ether extract and purified beta-carotene as the standard for mixed serum carotenoids, based on a method from 1963 [6]. Akbaraly and coworkers measured the absorbance of an ethanol / hexane blood plasma extract at 350, 450, and 550 nm and used an average molecular extinction coefficient and an “adequate equation” to adjust the absorbance at 450 nm for the absorbance at the other two wavelengths [7]. Generally, spectrophotometry of carotenoids has been reserved for pure carotenes or xanthophylls, but hasn’t been demonstrated to be useful for complex mixtures as seen in human blood serum [8].

This study establishes a robust and very useful correlation between the absorbance of an alcohol / heptane blood plasma extract and the concentration of total carotenoids determined by HPLC.

## Methods

### Subjects

Thirteen adults (5 men and 8 women) were recruited from a group that typically eats very high amounts of fruits and vegetables, “green drink” dietary supplements, and vegetable juice. Sixteen additional adults (8 men and 8 women) eating a more typical Western diet were recruited from a local community church group. Representation was sought across the spectrum of fruit and vegetable intakes for development of the assay, based on personal knowledge. No formal diet survey was collected from participants. All 29 subjects gave their informed consent before participating in the study. The protocol and informed consent process was reviewed and approved by WIRB (Seattle, WA).

### Materials

Chemicals. Capillary GC grade heptane was obtained from Sigma-Aldrich (St. Louis, MO). The alcohol was a common drugstore grade 70% isopropanol. Ethanol could also be used. Heparin, 5,000 IU/ml (Sagent Pharmaceuticals) was used as an anti-coagulant.

Equipment. The spectrometer used was a Jaz fiber optic spectrometer fitted with a pulsed xenon light source (Jaz-PX), XR grating from 200–1050 nm, a 10 μm entrance slit, 115 μm fiber optic light cable assemblies, and a temperature controlled 1-cm cuvette holder (Ocean Optics, Dunedin, FL). Data was captured via USB cable on a computer equipped with SpectraSuite, a Java-based, operating system independent spectroscopy software (Ocean Optics, Dunedin, FL). The use of the Jaz spectrometer system allows the user to see the entire visual spectrum instantly. This is beneficial for troubleshooting as well as carotenoid pattern recognition. Spectrum data was saved and converted to text format for analysis in Excel (Microsoft, Redmond, WA).

A semi-micro cuvette made with special optical glass was used for measurements (Cat No. 9/9-SOG-10, Starna Cells, Atascadero, CA). For this system with a Z dimension of 15 mm, the thicker base (9 mm instead of a standard 3 mm), along with one of the Teflon cuvette lids placed under the cuvette, allowed a reduced sample volume in the assay. A micro-centrifuge (Marathon 16 KM, Fisher Scientific, Pittsburg, PA) was used to spin down initial blood samples and a mini 6-place microfuge (Lab-Mini, Southwest Science, Bordentown, NJ) was used to spin the 0.5 ml microfuge tubes used for extractions. A circulating water bath was used to maintain constant temperature in the cuvette holder at 24°C. A bench top vortex mixer (Genie II) was used for mixing during extraction.

### Spectrophotometric assay method

Blood samples, between 1 and 1.5 ml each subject, were obtained by finger prick method using standard lancets. 30 IU of heparin were added to each sample for anticoagulant. Samples were spun 5 minutes at 2000 rpm (400x g) and plasma was removed for analysis. Carotenoid analysis was done in reduced light conditions. After initial analysis the samples were stored on dry ice.

A 50 μl aliquot of each sample was added to 50 μl of alcohol in a 0.5 ml microfuge tube and mixed. Then 150 μl of heptane was added and mixed by vortexing vigorously for 1 minute. Samples were then centrifuged for 1 minute and 145 μl of the heptane layer was removed and analyzed in the glass cuvette. The absorbance spectrum was corrected by adjusting for slight baseline shift, setting the absorbance at 550 nm to zero.

Heptane has been used for extraction of carotenoids from tomatoes in a study looking at in vitro approximation of absorption of carotenoids in vivo [9]. Heptane is not nearly as volatile as hexane, so it is much easier to work with, yet the molecular chain length is still very good for extracting the highly nonpolar carotenoids. Proteins and fats remain in the alcohol fraction, making a clean heptane layer containing only nonpolar molecules, including carotenoids, retinoids, and vitamin E. This method is not perturbed by hyperlipidemia or hemolysis because both fats and proteins remain in the alcohol layer.

Not all of the yellow color is removed from the plasma by this method. If acidified alcohol is used (>24 mM HCl, 0.2% 12 N HCl in alcohol) all of the pigments are extracted from the plasma into the heptane layer. However, bilirubin is also present in plasma and has a peak absorption at 450 nm, and is removed by acidification of the plasma. There may be other pigments as well in blood that are not carotenoids. The method with no more than 24 mM HCl works best for selectively removing carotenoids.

### Individual carotenoid analysis

Samples of plasma were stored on dry ice until shipment and analysis of carotenoids by Craft Technologies, Inc. (Wilson, NC), who specialize in fat-soluble vitamin and carotenoid analysis. Carotenoid concentrations were reported for six individual carotenoids and 4 isoforms. Samples were returned to Hallelujah Acres Foundation’s laboratory for further analysis by light spectrometry and refinement of assay conditions. All plasma samples were stored on dry ice.

## Results and discussion

### Lab values of individual carotenoids

Plasma total carotenoids ranged in concentration from 1.2 to 7.4 μM, as analyzed by HPLC. The range of total carotenoids in this study is much larger than the range (10-90^th^ percentile, 0.5 – 2.2 μM) in the general USA adult population [10]. This concentration range is typical of populations following a western diet. However, 5 of 62 population-based studies in a recent review reported upper partitions of subjects with plasma carotenoid concentrations between 4 and 7 μM [1], so this small sampling of 29 volunteers includes the full range of expected plasma carotenoid concentrations, with an over-sampling of the upper range.

In general, there were strong correlations between the individual carotenoid concentrations and the total carotenoid concentrations, as shown in Table 1. Both alpha- and beta-cryptoxanthin were not strongly correlated to total carotenoid concentrations. The USDA’s carotenoid database indicates that the richest dietary sources of cryptoxanthins are tangerines, chili powder, paprika, papaya, pumpkin, winter squash, and sweet red bell peppers [11]. Though dietary intake data was not collected in this study, from this list of foods one can see how a person could have a relatively high level of cryptoxanthins without an overall high intake of vegetables.

**Table 1:** Correlation of individual plasma carotenoids with total carotenoid concentration

**Carotenoid**	**Pearson correlation coefficient**
Lutein	0.739
Zeaxanthin	0.444
Cis-Lutein / Zeaxanthin	0.761
Alpha-Cryptoxanthin	0.298
Beta-Cryptoxanthin	0.515
Trans-Lycopene	0.091
Cis-Lycopene	0.098
Alpha-Carotene	0.830
Trans-Beta-Carotene	0.937
Cis-beta-carotene	0.935

The strongest correlation between the total carotenoid concentration and individual carotenoids was with trans-beta-carotene, with a correlation coefficient of 0.937. In fact, the total carotenoid concentration is almost a surrogate marker of beta-carotene. This is because the concentration of beta-carotene tends to be quite a bit higher than the other individual carotenoids, accounting for an average of 30 percent (range of 11–65 percent) of the total carotenoid concentration.

**Figure 1:**
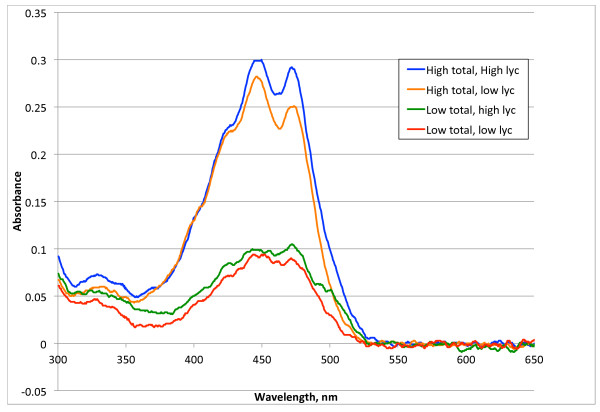
Spectrophotometric assay of carotenoid plasma extract.

The weakest correlation between total and individual carotenoids was with trans- and cis-lycopene. When the samples were examined by rank of total carotenoid concentrations, some samples with low total carotenoid concentrations actually had some of the highest lycopene concentrations. At low values of total carotenoids as much as fifty percent of some individual’s total carotenoids were lycopene (see Table 2). This pattern can also be recognized in the total carotenoid spectrum as well, as indicated in Figure 1. The visible spectrum of carotenoids typically has a shoulder near 425 nm, two peaks near 445–450 nm and near 470 nm, and a shoulder near 505 nm. In samples with high amounts of lycopene the peak at 470 nm is higher and the shoulder at 505 nm is more pronounced, especially when the total carotenoid concentration is low.

**Table 2:** A Carotenoid Health Index Based on Plasma Carotenoids and Health Outcomes

**Total carotenoids, μM**	**Alpha-carotene, %**	**Beta- carotene, %**	**Lycopene, %**	**Lutein, %**	**Beta-crypto- xanthin, %**	**Alpha-crypto- xanthin, %**	**Zea-xanthin, %**	**Cis-lutein / zeaxanthin, %**
1 to <1.5	7.1	17.0	43.8	13.4	7.8	2.3	5.7	2.9
1.5 to <2.5	9.8	24.1	33.2	12.8	10.0	2.3	5.1	2.8
2.5 to <4	13.0	36.9	16.1	16.1	8.9	1.6	4.8	2.7
4 to 10	16.0	45.9	11.1	15.6	4.9	0.8	3.2	2.5

Percent of total carotenoids given for each individual carotenoid for increasing concentration of total carotenoids.

There is a distinct shift in the percent of individual carotenoids as the total carotenoid concentration increased. As seen in Table 2, with increasing total carotenoids the mean percent of lycopene dropped from 43 to 11 percent, the mean percent of beta-carotene increased from 17 to 46 percent, and the mean percent of alpha-carotene increased from 7 to 16 percent. A similar trend of decreasing percentage of lycopene and increasing beta-carotene percentage with increasing total carotenoids was seen in a recent survey of plasma carotenoid concentrations in a representative population sample in the USA [10]. The trend in the CDC report was not so dramatic, probably because the range of total carotenoids was not so large as seen in this study.

### Lab value and spectrometric assay correlation

**Figure 2:**
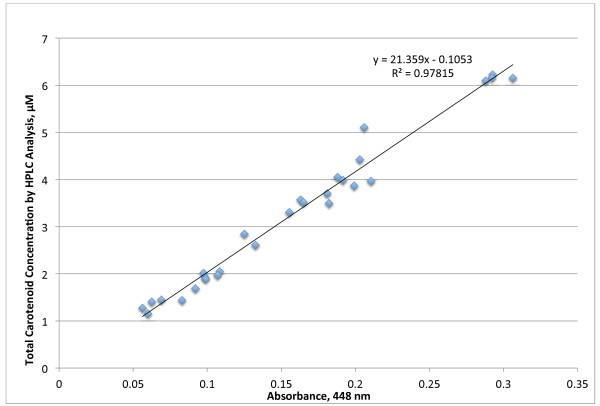
Correlation between spectrometer and HPLC assays of total plasma carotenoids.

There were 28 subjects that had enough plasma to compare HPLC and spectrometric assays. Figure 2 shows the correlation between estimated values of the total carotenoids, measured by the spectrometric assay, and the values determined by HPLC. The linear equation is total carotenoids = 21.359 · Abs 448 nm – 0.1053. The square of the Pearson correlation coefficient is 0.978, indicating a very usable correlation between these two assay techniques. The average CV was 6.5% for multiple analyses of these samples. The lowest detectable limit was around 0.3 μM and the assay is linear without dilution up to about 34 μM, well beyond the usual plasma range of total carotenoids.

Figure 2 indicates that the spectrometric assay is highly correlated with the HPLC analysis over a wide range of total carotenoid values and even with a variety of mixes of individual carotenoids as seen in Table 2. Some people had only 11 percent of total carotenoids as beta-carotene while others has as much as 65 percent. Lycopene ranged from 3 to 55 percent of total carotenoids. Yet the total carotenoids can be all fitted with one line. This makes this method robust and useful for quickly screening total carotenoid concentrations.

**Figure 3:**
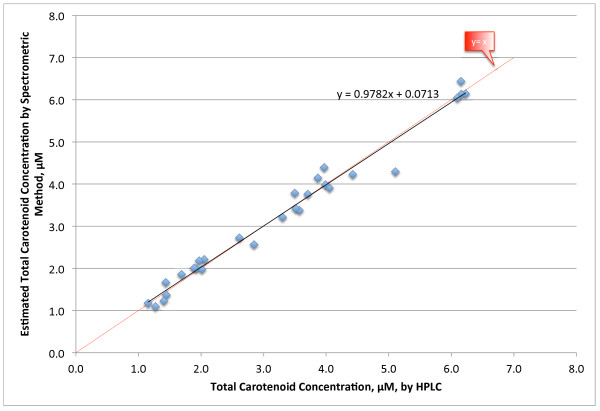
Total Carotenoid values by HPLC versus values calculated from the equation generated by the spectrometer assay.

**Figure 4:**
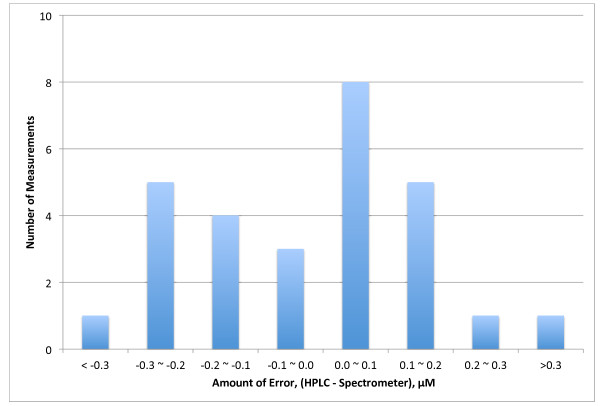
**Distribution of the error between the HPLC and spectrometric assay methods.** Values were determined by subtracting the spectrometric assay value from the HPLC value for each person.

Figure 3 gives a direct comparison between the total carotenoid values measured by HPLC and the total carotenoid concentration estimated by the equation above. This is another way of looking at the same data as in Figure 2. Except for a couple of points the plot lies close to the identity line, y = x. Figure 4 gives a histogram distribution of the errors between the HPLC and spectrometric methods. All but two of the measurements were within 0.3 μM of each other and 20 of 28 measurements lie within 0.2 μM of each other. The mean error was 0.17 μM (6.3%). The CV for this assay was 6.5%, so the error between the two methods is within the precision of the assay method. This error is likely to be less than day-to-day variations in a person’s plasma total carotenoid concentration.

### Absorbance versus integral over carotenoid absorption wavelengths

Analysis was completed to investigate whether any information could be added by looking at a wider range of the spectrum rather than a single wavelength. Integrals of the area under the absorbance versus wavelength plot were calculated. Various intervals were used, including wider ranges from 420–510 nm to narrow ranges from 435–475 nm. None of the correlations were as strong as simply using the absorbance at 448 nm. The output across the visible spectrum has some qualitative value, especially for troubleshooting an assay, but the peak absorbance at 448 nm is a better quantitative assessment of total carotenoid concentration.

### Calculated absorbance value

It is possible to calculate the expected absorbance value for each sample, knowing the published extinction coefficients for each carotenoid and the amount of each individual carotenoid present in each sample. Expected absorbance values were calculated from the HPLC values of individual carotenoids using published extinction coefficients [8]. The equation for the total carotenoid concentration based on this method is Total Carotenoids = 21.112 · Calculated Absorbance – 0.162, which is very close to the equation using the actual absorbance measurements.

**Figure 5:**
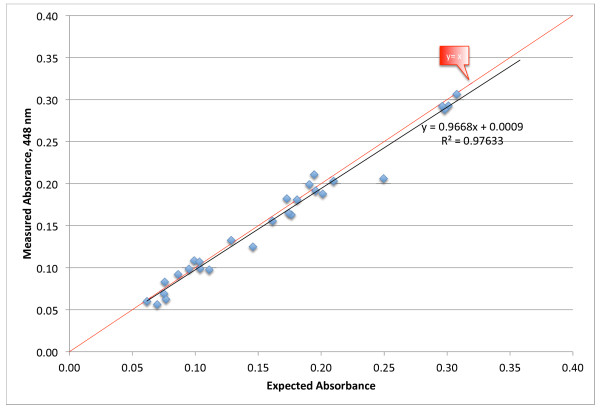
Expected (based on molecular extinction coefficients) versus actual spectrometer absorbance values.

Figure 5 shows the comparison of the measured values versus the expected values calculated from extinction coefficients of the individual carotenoids. The values fall very close to the identity line, y = x. The square of the Pearson correlation coefficient between the actual and expected absorbance values is 0.976, indicating a very close agreement between measured and expected values. A calculation using a different set of published extinction coefficients [2] gave the same result, with an average difference in expected absorbance of 0.00055 absorbance units.

The fact that the experimental and theoretical absorbance values are very similar implies that the extraction method was essentially complete and specific for carotenoids in the visible light spectrum range. The absorbance was not less than expected, as would be the case if the extraction was incomplete. Nor was the absorbance higher than expected, as would be the case if other compounds were extracted that absorbed light in the range of 400–500 nm. In fact, the leftover ethanol phase still has some yellow tint to it, indicating there are other substances, perhaps bilirubin and others, that could interfere with the assay. If all of the color is extracted, as can be done with acidified alcohol, the correlation between the absorbance and HPLC analysis of the samples is poor and unusable. The shape of the absorbance profile is also different, with a much higher peak at 450 nm (data not shown). The extraction cannot be guided by simply getting all of the yellow tint removed from the plasma.

There is another method of rapidly estimating body stores of carotenoids. This noninvasive method uses Raman spectroscopy to scan the palms of the hands. The Raman method has been used to show the value of carotenoid dietary supplements in increasing skin carotenoid concentrations. However, the correlation between skin carotenoid concentrations and plasma concentrations is only slightly better than correlations between plasma concentrations with diet records and food frequency questionnaires [12-14]. There are over one hundred reports on health outcomes and carotenoids based on plasma concentrations [1], while there are very few based on skin concentrations of carotenoids, so a carotenoid test based on plasma levels has solid scientific support. Plasma concentrations of carotenoids do vary a little, but as fat-soluble substances they reflect usual intake over a period of days, not just from the last meal. A minimally invasive, fast, inexpensive, accurate, direct assay of plasma carotenoids is much more insightful than skin carotenoid measurements.

The main limitation of this study would appear to be the small number of samples. However, the error between the HPLC measured carotenoids and the value calculated using the derived spectrophotometric equation was within the error of assay method, so more subjects would not necessarily improve either of these errors. There was good representation across the usual spectrum of carotenoid values in this study, including higher than typical levels. The results should be repeated and extended by others. In fact, many laboratories already have the equipment and could obtain anti-coagulant (heparin or EDTA both work well) and the solvents (any alcohol and heptane) to do this analysis. This assay could be rapidly used in many public health initiatives.

## Conclusions

The unique part of this study is that a spectrometric assay of carotenoids is standardized to the total carotenoid concentration analyzed by a gold standard method, HPLC. Previous methods have not provided this gold standard comparison. As such, this assay method is a robust, inexpensive, fast, and accurate measurement of total plasma carotenoids. This rapid assay, when coupled with the carotenoid health index, may be useful for nutrition intervention studies, population cohort studies, and public health interventions.

### Availability and requirements

Project name: Rapid Assay of Total Carotenoids

Project homepage: None.

Operating System: None.

Other requirements: Spectrophotometer

License: None.

Any restriction to use by non-academics: None.

### Availability of supporting data

The data set supporting the results of this article is included within the article.

## Abbreviations

HPLC: High Performance Liquid Chromatography; USDA: United States Department of Agriculture; GC: Gas chromatography; WIRB: Western Institutional Review Board.

## Competing interests

Michael Donaldson is a research scientist contracted by Hallelujah Acres to conduct investigations pertaining to the Hallelujah Diet. Funding for this research was provided by Hallelujah Acres.

## Authors’ contribution

MD designed this study, was responsible for data collection and interpretation, and preparation of this manuscript.

## References

[B1] Donaldson MS A Carotenoid Health Index Based on Plasma Carotenoids and Health Outcomes Nutrients 2011 3 1003 1022 10.3390/nu3121003 22292108 PMC3260489

[B2] Craft N Brown E Smith J Effects of storage and handling conditions on concentrations of individual carotenoids, retinol, and tocopherol in plasma Clin Chem 1988 34 44 48 [published erratum appears in Clin Chem 1988 Jul;34(7):1505] 3338183

[B3] Palmer LS Eckles CH Carotin–The principal natural yellow pigent of milk fat: Its relations to plant carotin and the carotin of the body fat, corpus luteum and blood serum J Biol Chem 1914 17 191 210

[B4] Connor CL Studies on lipochromes J Biol Chem 1928 77 619 626

[B5] Sahyoun NR Jacques PF Russell RM Carotenoids, vitamins C and E, and mortality in an elderly population Am J Epidemiol 1996 144 501 511 10.1093/oxfordjournals.aje.a008957 8781466

[B6] Neeld JB Pearson WN Macro- and Micromethods for the Determination of Serum Vitamin A using Trifluoroacetic Acid J Nutr 1963 79 454 462 13937886 10.1093/jn/79.4.454

[B7] Akbaraly TN Favier A Berr C Total Plasma Carotenoids and Mortality in the Elderly: Results of the Epidemiology of Vascular Ageing (EVA) Study Br J Nutr 2009 101 86 92 10.1017/S0007114508998445 18507882

[B8] Scott KJ Wrolstad RE, Acree TE, Decker EA, Penner MH, Reid DS, Schwartz SJ, Shoemaker CF, Smith D, Sporns P, Hoboken NJ Detection and Measurement of Carotenoids by UV/VIS Spectrophotometry Current Protocols in Food Analytical Chemistry 2001 USA: John Wiley & Sons, Inc

[B9] van het Hof KH de Boer BCJ Tijburg LBM Lucius BRHM Zijp I West CE Hautvast JGAJ Weststrate JA Carotenoid Bioavailability in Humans from Tomatoes Processed in Different Ways Determined from the Carotenoid Response in the Triglyceride-Rich Lipoprotein Fraction of Plasma after a Single Consumption and in Plasma after Four Days of Consumption J Nutr 2000 130 1189 1196 10801917 10.1093/jn/130.5.1189

[B10] CDC National Report on Biochemical Indicators of Diet and Nutrition - Vitamins A and E and Carotenoids 2008 Center for Disease Control and Prevention: Atlanta, GA

[B11] U.S. Department of Agriculture, Agricultural Research Service USDA National Nutrient Database for Standard Reference, Release 23 2010 Beltsville, MD: Nutrient Data Laboratory

[B12] Mayne ST Cartmel B Scarmo S Lin H Leffell DJ Welch E Ermakov I Bhosale P Bernstein PS Gellermann W Noninvasive assessment of dermal carotenoids as a biomarker of fruit and vegetable intake Am J Clin Nutr 2010 92 794 800 10.3945/ajcn.2010.29707 20685953 PMC3133234

[B13] Smidt CR Burke DS Nutritional significance and measurement of carotenoids Curr Topics Nutraceut Res 2004 2 79 91

[B14] Zidichouski JA Mastaloudis A Poole SJ Reading JC Smidt CR Clinical validation of a noninvasive, Raman spectroscopic method to assess carotenoid nutritional status in humans J Am Coll Nutr 2009 28 687 693 20516269 10.1080/07315724.2009.10719802

